# Co-Stimulation of PAFR and CD36 Is Required for oxLDL-Induced Human Macrophages Activation

**DOI:** 10.1371/journal.pone.0036632

**Published:** 2012-05-03

**Authors:** Francisco J. O. Rios, Mariana M. Koga, Matheus Ferracini, Sonia Jancar

**Affiliations:** Department of Immunology, Institute of Biomedical Sciences, University of São Paulo, Sao Paulo, Brazil; Centro Cardiologico Monzino, Italy

## Abstract

The oxidative process of LDL particles generates molecules which are structurally similar to platelet-activating factor (PAF), and some effects of oxidized LDL (oxLDL) have been shown to be dependent on PAF receptor (PAFR) activation. In a previous study, we showed that PAFR is required for upregulation of CD36 and oxLDL uptake. In the present study we analyzed the molecular mechanisms activated by oxLDL in human macrophages and the contribution of PAFR to this response. Human adherent monocytes/macrophages were stimulated with oxLDL. Uptake of oxLDL and CD36 expression were determined by flow cytometry; MAP kinases and Akt phosphorylation by Western blot; IL-8 and MCP-1 concentration by ELISA and mRNA expression by real-time PCR. To investigate the participation of the PI3K/Akt pathway, Gαi-coupled protein or PAFR, macrophages were treated with LY294002, pertussis toxin or with the PAFR antagonists WEB2170 and CV3988, respectively before addition of oxLDL. It was found that the addition of oxLDL to human monocytes/macrophages activates the PI3K/Akt pathway which in turn activates the MAPK (p38 and JNK). Phosphorylation of Akt requires the engagement of PAFR and a Gαi-coupled protein. The upregulation of CD36 protein and the uptake of oxLDL as well as the IL-8 production are dependent on PI3K/Akt pathway activation. The increased CD36 protein expression is dependent on PAFR and Gαi-coupled protein. Transfection studies using HEK 293t cells showed that oxLDL uptake occurs with either PAFR or CD36, but IL-8 production requires the co-transfection of both PAFR and CD36. These findings show that PAFR has a pivotal role in macrophages response to oxLDL and suggest that pharmacological intervention at the level of PAFR activation might be beneficial in atherosclerosis.

## Introduction

Accumulation of modified low-density lipoprotein (LDL), such as oxidized LDL (oxLDL), in the arterial wall, and the recruitment of monocytes to the subendothelial space are known to be the main early events in the development of atherosclerosis [1]. Macrophages express receptors that bind and internalize oxidized forms of LDL. It is known that CD36 receptor expression is not regulated by intracellular levels of cholesterol, and is increased in atherosclerotic lesions because of auto-regulation by components of the oxLDL particles [2]. This uncontrolled uptake leads to differentiation of the macrophage into foam cells that play a critical role in development and progression of the atherosclerotic plaque [3].

A number of laboratory studies have provided compelling evidence that CD36 is one of the main scavenger receptors involved in the uptake of oxLDL by monocytes/macrophages. Its deficiency greatly reduced the uptake of oxLDL and atherosclerotic lesions in mice models [4], [5]. Moreover, monocytes from individuals lacking CD36, or experiments using functional blockage with antibodies, decreased oxLDL uptake by about 50% [4].

The mechanism of oxLDL-induced foam cell formation during the atherosclerotic process has been under intense investigation, and appears to be dependent on several membrane and nuclear receptors that might act, either independently or synergistically, to mediate the cell response [1], [6]. The uptake of oxLDL leads to activation of a MAPK pathway that exerts a crucial role in foam cell formation and is essential for IL-8 production, which mediates the cell recruitment to the plaque site, accelerating the atherogenic process and arterial thickness [7]. Moreover, MAPK activation has anti-apoptotic effects, contributing to macrophage proliferation and foam cell survival in the atherosclerotic plaque [8].

The oxidative process of LDL particles generates molecules which are structurally similar to the lipid mediator platelet-activating factor (PAF), and some effects of oxLDL have been shown to be dependent on PAFR activation [9], [10]. Although these PAF-like compounds are less potent than PAF in binding to PAFR, they are considerably more abundant [9]. Our previous study revealed that oxLDL activates MAPK (ERK1/2 and JNK) and increases the expression of CD36 in human cells by PAFR-dependent mechanisms. Moreover we have shown that uptake of oxLDL is also dependent on PAFR [11]. Since the main receptor that mediates the oxLDL uptake by macrophages is CD36, which has a very short intracytoplasmatic tail that hardly activates intracellular signaling pathways, we hypothesized that the effect of oxLDL might be caused by co-stimulation of CD36 and PAFR [11]. In the present study, we confirmed this hypothesis by demonstrating the requirement of both receptors for macrophage response to oxLDL and further investigated the molecular mechanisms involved.

## Results

### oxLDL activates PI3K/Akt pathway in a PAFR and Gαi-coupled protein- dependent manner

**Figure 1:**
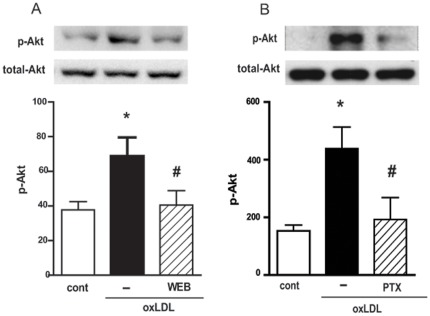
oxLDL activates the PI3k/Akt pathway in a PAFR and Gαi coupled protein-dependent manner. Adherent monocytes/macrophages were treated with WEB2170 (50 µM, 30 min) (A) or PTX (600 ng/mL, 18 h) (B) and stimulated with oxLDL (30 µg/mL) for 10 min. Western blot analysis of cell lysates were performed using antibodies to phosphorylated and non-phosphorylated forms of Akt. Data are presented as mean ± SEM of four donors. Protein expression was quantified by the AlphaEaseFC™ software V3.2 beta (Alpha Innotech). The autoradiographs show one representative experiment. * p<0.05 comparing oxLDL-stimulated with the non-stimulated cells (cont). **#** p<0.05 comparing cells treated with WEB2170 or PTX with non-treated cells.

Increasing evidence is emerging that the activation of PI3K and its downstream effector Akt plays a role in macrophage activation induced by oxLDL [12]. We therefore investigated whether PAFR and a Gαi-coupled protein are involved in this activation. Human macrophages were pre-treated with WEB2170 for 30 min or with PTX for 18 h and then stimulated with oxLDL. We found that pre-treatment with a PAFR antagonist and PTX inhibited the Akt phosphorylation (90% and 86% inhibition respectively) induced by oxLDL (Fig. 1A and 1B).

**Figure 2:**
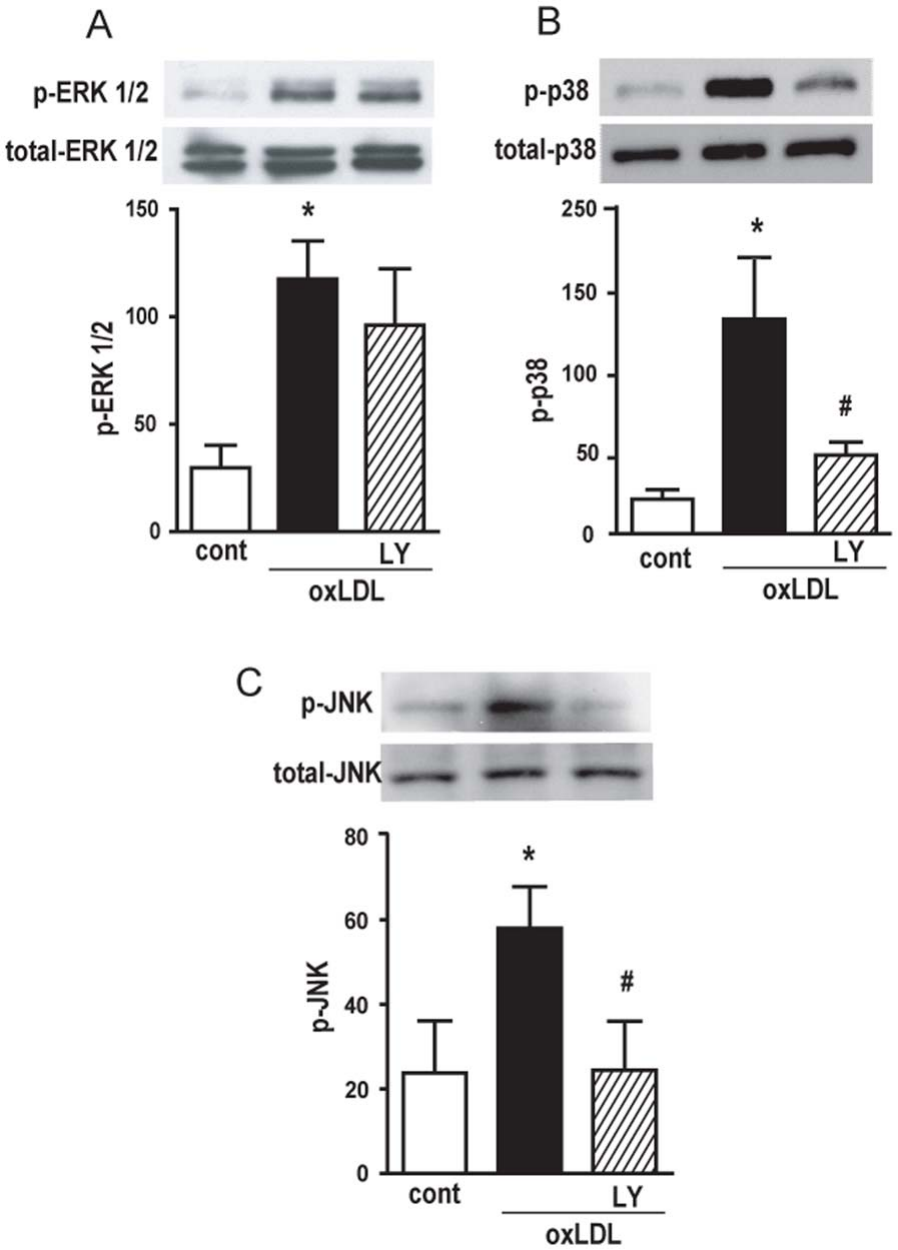
PI3k/Akt pathway is required for p38 and JNK MAPK activation induced by oxLDL. Adherent monocytes/macrophages were treated with LY294002 (10 µM, 30 min) and then stimulated with oxLDL (30 µg/mL) for 10 min. The MAPK Western blot analysis of cell lysates were performed using antibodies to phosphorylated and non-phosphorylated forms of ERK1/2, p38 and JNK (A, B and C, respectively). The autoradiographs show one representative experiment. Graph data are presented as mean ± SEM of four donors. Protein expression was quantified by the AlphaEaseFC™ software V3.2 beta (Alpha Innotech). * p<0.05 comparing oxLDL-stimulated with the non-stimulated cells (cont). **#** p<0.05 comparing cells treated with LY294002 with non-treated cells.

In a previous study we showed that oxLDL induces the activation of the MAPK pathway and that this effect is PAFR-dependent [11]. We then tested whether PI3K activation is required for MAPK phosphorylation. Human macrophages were pre-treated with PI3K inhibitor LY294002 and stimulated with oxLDL. It was found that oxLDL induced the phosphorylation of ERK1/2, JNK and p38 (Fig. 2). Figures 2B and 2C demonstrate that pre-treatment with LY294002 inhibited the phosphorylation of p38 and JNK (75% and 100% inhibition, respectively). However, the phosphorylation of ERK1/2 was not affected by the inhibitor of PI3K (Fig. 2A). These data clearly demonstrate that, although oxLDL induced the phosphorylation of ERK1/2, p38 and JNK, only the phosphorylation of p38 and JNK was mediated by PI3K activation.

### Activation of the PI3K/Akt pathway is required for macrophage responses to oxLDL: upregulation of CD36, oxLDL uptake and IL-8 production

**Figure 3:**
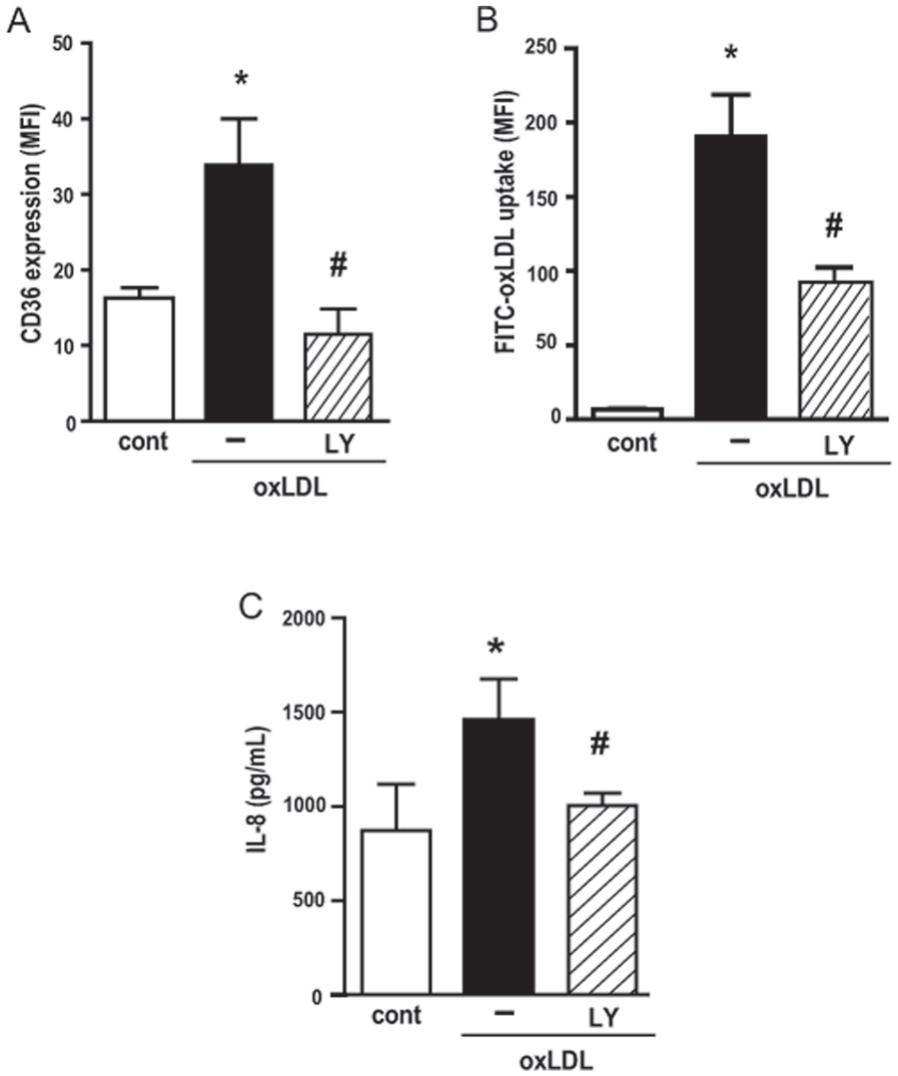
PI3K/Akt pathway is required for oxLDL induced upregulation of CD36 expression (A) oxLDL uptake (B) and IL-8 production (C). Adherent human monocytes/macrophages were treated with LY294002 (10 µM, 30 min), and then stimulated with oxLDL (30 µg/mL) for 24 h. The expression of CD36 was measured by FACS and IL-8 production by ELISA. For the uptake evaluation, stimulated cells were washed and incubated with FITC-oxLDL for 1 h and uptake was measured by FACS. Data are presented as mean ± SEM of the mean fluorescence Intensity of five donors. * p<0.05 comparing oxLDL stimulated with non-stimulated cells (cont). # p<0.05 comparing cells treated with LY294002 with non-treated cells.

We then investigated whether PI3K was necessary for the response of macrophages to oxLDL, namely the upregulation of CD36 expression, oxLDL uptake and IL-8 production. To this purpose, human macrophages were pre-treated with LY294002 for 30 min before addition of oxLDL. We found that LY294002 abolished the upregulation of the CD36 expression induced by oxLDL (Fig. 3A) and significantly inhibited the FITC-oxLDL uptake (Fig. 3B). Figure 3C shows that the treatment with PI3K inhibitor also markedly decreased the production of IL-8 by human macrophages.

### CD36 expression induced by oxLDL is dependent on PAFR and Gαi- coupled GPCR

**Figure 4:**
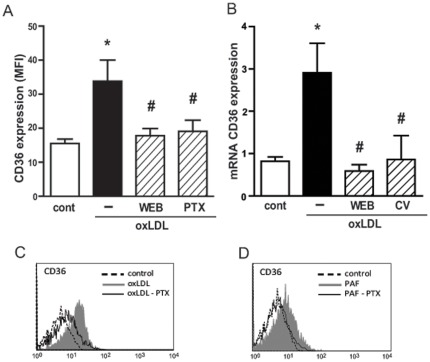
PAFR and Gαi-coupled protein is required for upregulation of CD36 protein and mRNA expression. Adherent human monocytes/macrophages were treated with WEB2170 (50 µM, 30 min) or with pertussis toxin (PTX - 600 ng/mL, 18 h) and then stimulated with oxLDL (30 µg/mL) or PAF (10^−6^ M). The expression of CD36 was measured by FACS after 24 h (A, C and D) and the mRNA levels after 5 h (B). Data are presented as mean ± SEM of the mean fluorescence intensity of five donors (A–B) or one representative experiment (C–D). * p<0.05 comparing oxLDL stimulated with non-stimulated cells (cont). # p<0.05 comparing cells treated with PTX, WEB2170 or CV3988, with non-treated cells.

Figure 4A shows that the incubation of human macrophages with oxLDL increased CD36 protein expression, and this effect was abrogated by previous treatment with the PAFR antagonist confirming our previous data [11]. This effect was also observed at mRNA levels, where two molecularly unrelated PAFR antagonists (WEB2170 and CV3988) inhibited the oxLDL-induced CD36 mRNA expression (Fig. 4B). Because the PAFR is a G-coupled receptor, we sought to investigate the type of Gα-coupled protein involved in the CD36 expression induced by oxLDL. Thus, human macrophages were pre-treated with the Gαi inhibitor pertussis toxin (PTX) (600 ng/mL) for 18 h and stimulated with oxLDL (30 µg/mL) or PAF (10^−6^ M). The expression of CD36 was evaluated after 24 h by FACS. Figures 4A and 4C show that the pre-treatment with PTX inhibited the expression of CD36 induced by oxLDL (78% inhibition). When the agonist PAF was added to the macrophages, it increased the expression of CD36 and this effect was also abrogated by PTX treatment (Fig. 4D). PTX did not affect the cell viability, measured by MTT assay, which was around 98% in both treated and non-treated cells (data not shown).

### oxLDL induces IL-8 and MCP-1 chemokines in a PAFR-dependent manner

**Figure 5:**
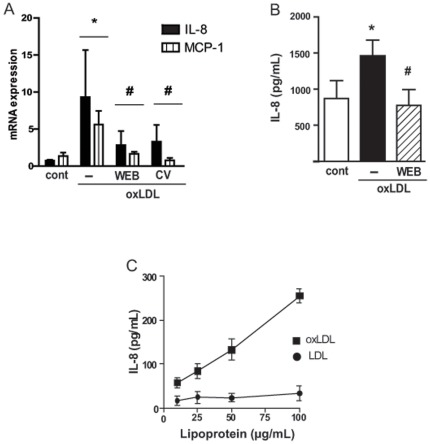
oxLDL induces IL-8 and MCP-1 in a PAFR-dependent manner. Adherent human monocytes/macrophages were treated with WEB2170 (50 µM) or CV3988 (10 µM) for 30 min and stimulated with oxLDL (30 µg/mL). The mRNA levels for MCP-1 and IL-8 were evaluated after 5 h (n = 4) (A). The IL-8 concentration was measured in the supernatants after 24 h (n = 3) (B). THP-1 cells stimulated with different concentrations of oxLDL or LDL, the IL-8 concentration was measured after 24 h (C). Data are presented as mean ± SEM. * p<0.05 comparing oxLDL stimulated with non-stimulated cells (cont). **#** p<0.05 comparing cells treated with WEB2170 or CV3988 with non-treated cells.

We found that oxLDL stimulates IL-8 and MCP-1 mRNA expression in human monocytes/macrophages and this requires the PAFR activation since the antagonists WEB2170 and CV3988 reduced mRNA levels for those chemokines (Fig. 5A). The production of IL-8 protein was also dependent on PAFR (Fig. 5B). Moreover, only oxLDL and not the non-modified LDL was able to induce IL-8 production in a monocytic cell line, THP-1, in a dose response-dependent manner.

These results show that PAFR is involved in macrophage activation by oxLDL to induce chemokine production.

### The uptake of oxLDL can occur through PAFR and CD36 separately but co-stimulation of both receptors is required to induce IL-8 production

**Figure 6:**
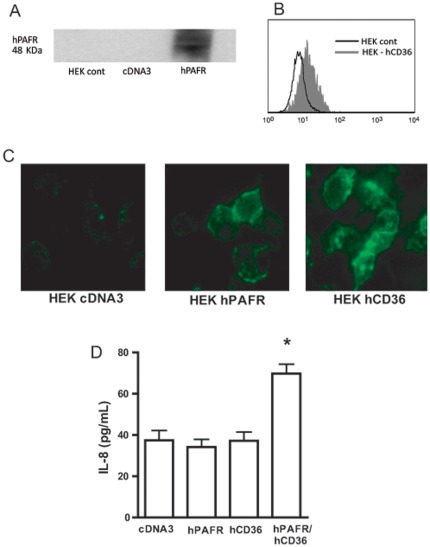
CD36 and PAFR cooperatively mediate FITC-oxLDL uptake and IL-8 production. HEK 293t cells were transiently transfected with hCD36 and/or hPAFR, as described in methods (A–B). Transfected cells were incubated with FITC-oxLDL (30 µg/mL) for 1 h at 37°C. Cells were washed and fixed with paraformaldehyde 4%. Fluorescence was visualized by microscopy (C). Transfected cells were stimulated with oxLDL (30 µg/mL) and the IL-8 concentration in the supernatant was measured after 24 h (D). * p<0.05 comparing with cDNA3 plasmid control transfected cells.

HEK293t cells were transiently transfected with plasmids encoding hPAFR cDNA and hCD36 cDNA (Fig. 6A and 6B). The involvement of PAFR and/or CD36 in the oxLDL uptake was studied by incubation of HEK293t-hPAFR or HEK293t-hCD36 with FITC-oxLDL, and the uptake was visualized by fluorescence microscopy. Figure 6C shows that, under the experimental conditions employed, FITC-oxLDL was taken up by cells expressing CD36 and, to a lesser extent, by PAFR. In order to investigate whether both receptors were required for the IL-8 production induced by oxLDL, HEK293t-transfected cells were stimulated with oxLDL and after 24 h the IL-8 production was evaluated in the supernatant (Fig. 6D). It was found that oxLDL induced IL-8 production only in HEK293t cells co-transfected with PAFR, in combination with CD36. IL-8 was not detected in single transfected cells. These data indicated that, although the uptake of oxLDL can occur separately through each receptor, the co-stimulation of both receptors is needed to induce IL-8 production.

## Discussion

**Figure 7:**
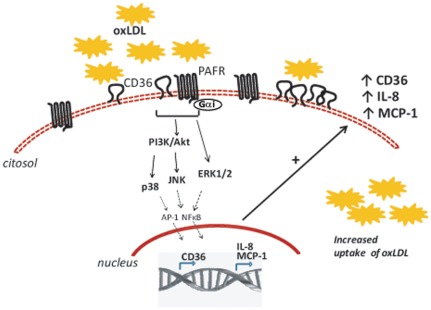
Based on these results we conclude that the addition of oxLDL to human monocytes/macrophages activates the PI3K/Akt pathway which in turn activates the p38 and JNK MAPK. Phosphorylation of Akt requires engagement of PAFR and a Gαi-coupled protein. This should lead to NFκB and/or AP-1 activation and gene transcription. We found increased expression of CD36, IL-8 and MCP-1 mRNA in cells stimulated with oxLDL in a PAFR-dependent manner. Moreover, we observed increased expression of CD36 protein on macrophage membrane and increased uptake of oxLDL by these cells upon exposure to oxLDL which were also dependent on PAFR. By transfecting CD36 and PAFR to HEK 293t cells, we found that whereas each receptor *per se* was able to induce oxLDL uptake, both receptors were required for IL-8 production. These results point to an important role for PAFR in atherosclerosis by promoting foam cells formation because of the increased uptake of oxLDL and also by contributing to macrophage infiltration due to IL-8 and MCP-1 production. Thus, antagonists of PAFR might be a promising target for atherosclerosis treatment. Dashed lines represent data obtained from the literature [21].

In a previous study we showed that PAFR is required for upregulation of CD36 expression and oxLDL uptake by MAPK pathway activation [11]. Here we showed that the addition of oxLDL to human monocytes/macrophages activates the PI3K/Akt pathway which in turn activates the p38 and JNK MAPK. Phosphorylation of Akt requires the engagement of PAFR and a Gαi-coupled protein. This leads to increased expression of CD36, IL-8 and MCP-1 mRNA and is also PAFR-dependent (Fig. 7). By transfecting CD36 and PAFR to HEK 293t cells, we found that whereas each receptor *per se* was able to induce oxLDL uptake both receptors were required for IL-8 production.

It has been reported that oxLDL activates several receptors expressed in macrophages and contributes to the inflammatory response present in early and advanced atherosclerotic lesions [13]. This is possibly because oxLDL is not a single particle but a number of oxidized compounds that might interact with different receptors and promote various intracellular activation pathways [10], [14]. These multiple effects explain why the detailed mechanism involved in macrophage activation induced by oxLDL, and the intracellular pathway to foam cell formation are not fully understood. Therefore, it is important to characterize the signals induced by oxLDL in the cells that participate in the atherosclerotic lesion, and to determine which signals are relevant in the pathogenesis of atherosclerosis. It has been shown that MAPK activation is required for foam cell formation and atherosclerotic lesion in animal models [15], [16]. Our results using human cells show that the phosphorylation of p38 and JNK induced by oxLDL, which is required for the uptake of oxLDL, is dependent on PI3K/Akt activation. Moreover, a PAFR antagonist and a Gαi inhibitor decreased the phosphorylation of Akt, which is known to be a downstream target protein for PI3K and a critical player in promoting the macrophage survival by virtue of its anti-apoptotic mechanisms [17]. These results suggest that the activation of PAFR and Gαi-coupled receptor, induced by the interaction of oxLDL with macrophages, induces the PI3K/Akt activation pathway and MAPK phosphorylation which are necessary for the upregulation of CD36 expression, oxLDL uptake and IL-8 production. One possible mechanism responsible for CD36 expression involves the activation of PPARγ [2] and Nrf2 [18] which have been described as the main pathways and might be a downstream pathway for PI3K/Akt activation [17]. However, the importance of the PI3k-Akt-PPARγ-CD36 or PI3k-Akt-Nrf2-CD36 pathways has never been addressed.

We previously reported that ERK1/2 inhibition reduced the expression of CD36 [11]. In this work the PI3K inhibitor reduced the expression of CD36 without affecting the ERK1/2 phosphorylation. Together these results are very interesting and we can speculate that the downstream molecules from ERK1/2 and Akt pathways might interact synergistically to induce CD36 expression. Corroborating this hypothesis, it was demonstrated that ERK1/2 and Akt inhibitors impair cell proliferation and tumor growth synergistically even though Akt was not involved in ERK1/2 phosphorylation [19].

Several pro-inflammatory cytokines and chemokines related to atherosclerosis progression have been observed in macrophages stimulated by oxLDL and compounds present in the oxLDL particle [20]. We found that macrophages stimulated with oxLDL produced high IL-8, an effect inhibited by the PAFR antagonist and PI3K inhibitor. The production of IL-8 is dependent on MAPK phosphorylation, which activates the nuclear factor AP-1 and stabilizes IL-8 mRNA [21]. This chemokine was first described as a mainly neutrophil chemoattractant, and probably because of this there is a lack of information regarding the role of IL-8 in atherogenesis. It is now known, however, that IL-8 plays an early and important role in recruitment of monocytes to atherosclerotic lesions [22]. Moreover, chronic IL-8 production also triggers hyperplasia, leading to vessel wall thickening and acceleration of the atherogenic process [7]. Our data suggest that the PAFR is required not only for oxLDL uptake but also for IL-8 and MCP-1 production, which are important for the recruitment of monocytes to the subendothelial space. More importantly, IL-8 has recently been indicated as a potential marker of atherosclerotic disease [23].

Regarding the involvement of PAFR in atherosclerosis, it is known that foam cells produce high amounts of PAF [24], [25]. Only one study has shown that treatment with PAFR antagonists reduced the formation of fatty streaks lesions, which are the first stage in atherosclerosis development [26]. However, the role of PAFR in mechanisms related to advanced atherosclerotic plaque has never been addressed. The scavenger receptor CD36 has a very short intracytoplasmatic tail and there are still conflicting data regarding the mechanisms of signal transduction mediated by this receptor. CD36 does not appear to signal on its own in response to stimuli but it has been shown that, depending on the structural characteristics of the ligand, it might collaborate with TLR2, TLR4 and TLR6, resulting in different cell responses [13], [27]. oxLDL is a complex particle, formed by several oxidized phospholipids that interact with CD36 and PAFR and little is known about the influence of this interaction on cell activation. To address these questions, we used human cell-based models in which CD36 and/or PAFR were over-expressed in HEK293t cell lines. This cell line was selected because of non-existent levels of receptors, allowing dissection of direct CD36- and PAFR-mediated signaling. The obtained results showed that both receptors, PAFR and CD36, are able to bind the oxLDL but only the co-stimulation of CD36 and PAFR by oxLDL was able to induce the production of IL-8. These data suggest that although PAFR and CD36 can independently promote the uptake of oxLDL, only the co-stimulation of both receptors is able to transduce intracellular signaling for IL-8 production, thus indicating a crosstalk between receptors for PAF and oxLDL. Our study did not show at what level the interaction between CD36 and PAFR occurs. If these receptors are stimulated in parallel, or if oxLDL can recruit both classes of receptors to specific membrane microdomains called lipid rafts, then this would allow these receptors to be in close proximity and induce a favorable environment for their association. In our study, we focused on PAFR and CD36 but several innate immune receptors have been shown to mediate cell activation by oxLDL [13], [27]. It is therefore possible that other receptors might have a role in this assembly and in increasing cell activation.

In conclusion, the intracellular signaling pathways activated by oxLDL are dependent on PAFR and play a pivotal role in mediating the oxLDL uptake and consequently have functions in foam cell formation. Additionally, this study shows that oxLDL interacts with CD36 and PAFR to induce IL-8 and MCP-1, which are involved in the recruitment of monocytes and may contribute to inflammatory processes. Thus, PAFR could become a promising target for atherosclerosis treatment.

## Materials and Methods

### Materials

Fetal calf serum (FCS), RPMI-1640, L-glutamine and HEPES acid were purchased from Gibco (Long Island, NY, USA); penicillin, streptomycin, aprotinin, leupeptin, benzamidin, chloramphenicol, protease inhibitor cocktail, PMSF, pertussis toxin from Bordetella pertussis, MTT (methylthiazolyldiphenyl-tetrazolium bromide) and the Ficoll Hypaque gradient: Histopaque®-1077 from Sigma-Aldrich (St. Louis, MO, USA); the PI3K inhibitor LY294002 and the PAFR human polyclonal antibody from Cayman Chemical (Ann Arbor, Michigan, USA); the monoclonal phycoerythrin (PE)-anti-hCD36 and isotype control PE-mouse IgM from BD Biosciences (Franklin Lakes, NJ, USA); the monoclonal antibodies to phosphorylated and non-phosphorylated forms of ERK1/2, p38, JNK, Akt and anti-rabbit IgG-HPR from Cell Signaling Technology (Beverly, MA, USA); the Qiagen plasmid midi Kit from Qiagen Inc. (Valencia, CA, USA); the Lipofectamine™ 2000 from Invitrogen by Life Technologies (Carlsbad, CA, USA); the ECL reagent kit and the nitrocellulose membrane for western blot from GE Healthcare (Uppsala, Sweden); the Ficoll–Hypaque from Amersham-Pharmacia Biotech (Uppsala, Sweden); the BCA kit from Thermo Scientific (Rockford, IL, USA); PAFR antagonists: CV3988 was purchased from Biomol (Enzo Life Sciences, New York, USA) and WEB2170 was kindly supplied by Boehringer Ingelheim (Pharma KG, Biberach, Germany).

### Purification and oxidation of LDL

The study was approved by the ethics committee of the Institute of Biomedical Sciences, University of Sao Paulo. Blood was collected from normolipidemic volunteers and plasma was obtained after centrifugation at 1,000 *g*, 4°C, for 15 min in the presence of EDTA 1 mg/mL. Thereafter, we added benzamidine (2 mM), gentamicin (0.5%), chloramphenicol (0.25%), PMSF (phenyl-methyl-sulfonyl-fluoride) (0.5 mM), and aprotinin (0.1 units/mL). LDL (1.019–1.063 g/mL) was isolated by sequential ultracentrifugation (100,000 *g*, 4°C), using a P90AT-0132 rotor (CP70MX ultracentrifuge; Hitachi Koki Co., Ltd, Tokyo, Japan), dialyzed (4°C) against PBS, pH 7.4 containing 1 mM EDTA, filtered (0.22 µm) and stored at 4°C. The protein concentration was determined by the BCA kit. Part of the LDL was dialyzed overnight against EDTA-free PBS, followed by incubation with CuSO_4_ (20 µM per mg of LDL protein/18 h/37°C). The oxidation was stopped by the addition of EDTA 1 mM. This procedure has been previously standardized and results in completely oxidized LDL [28].

### Cell culture

Peripheral blood mononuclear cells (PBMC) were separated by a Ficoll–Hypaque gradient (density 1.077 g/mL) according to the manufacturer's instructions, resuspended and cultured in RPMI-1640 supplemented with 5% FCS, 15 mM HEPES, 2 mM L-glutamine, 100 U/mL penicillin and 100 µg/mL streptomycin. Cell cultures were maintained in a humidified atmosphere containing 5% CO_2_ at 37°C. After 1 h, the non-adherent cells were washed out with warm PBS and the adherent monocytic cells were cultured in RPMI/10% FCS for 72 h. The monocytic cell line THP-1 cells were cultured in RPMI/10% FCS. One day before the experiments, the medium was changed for RPMI/2% FCS.

### Flow cytometry for CD36 expression

Macrophages were cultured in the presence of oxLDL for 24 h. The PAFR antagonists WEB2170 or the inhibitors of PI3 kinase (LY294002) were added to the cell cultures 30 min before the addition of oxLDL. After the incubation time, the cell culture medium was replaced with cold PBS and plates were put on ice for 30 min, after that, the cells were scraped with a rubber policeman cell, centrifuged at 300× g for 10 min, washed and resuspended in staining buffer (PBS, FCS 1%, sodium azide 0.1%) containing the labeled monoclonal antibodies, PE-conjugated anti-human CD36 or the isotype control, diluted 1∶100. The expression of CD36 was evaluated in a flow cytometer (FACS Canto II - Becton BD Biosciences) and the data were analyzed by the software Summit® V4.3 (DakoCytomation). During the acquisition, the doublets were excluded using gates in FSC-A *vs* FSC-H. The autofluorescence of macrophages was removed at the beginning of the analyses.

### Uptake of oxLDL

LDL was labeled with FITC, as described previously [29], [30]. The FITC concentration in LDL was determined by spectroscopy against FITC standard solution at 495 nm. The F/P (fluorochrome/protein) molar ration was calculated as described and admitted in the range of 2.4 to 3 [31]. FITC-LDL was oxidized by CuSO_4_ (20 µM per mg of LDL protein; 18 h; 37°C). To investigate the role of the PI3K in the uptake of oxLDL, monocytes/macrophages were treated with LY294002 (10 µM) for 30 min, then incubated with oxLDL (30 µg/mL). After 24 h, the cells were washed twice with RPMI/5% FCS to remove the oxLDL from the supernatant and incubated overnight in RPMI/5% FCS. This procedure was performed to allow the upregulation of CD36 in the plasma membrane. The uptake assay was performed by incubation with FITC-oxLDL for 1 h at 37°C. Cells were washed with cold PBS and fixed with 2% formaldehyde. The uptake of FITC-oxLDL was evaluated by flow cytometry using a FACS Canto II (Becton BD Biosciences) and analyzed using software Summit® V4.3 (DakoCytomation).

### Western blot for ERK1/2, p38-MAPK, JNK and Akt

Human monocytes/macrophages (2×10^6^ cells/ml) were pre-treated with LY294002 (10 µM) for 30 min, then treated with oxLDL for 10 min. Cells were washed with cold PBS and protein lysates were obtained by homogenizing cells in lysis buffer (1% Nonidet® P40, 50 mM Tris, pH 8.0, 150 mM NaCl, SDS 0.1%) supplemented with a protease inhibitor cocktail and phosphatase inhibitors (10 mM sodium fluorate and 1 mM sodium orthovanadate). Protein concentrations were determined using with the BCA assay kit. Equal amounts of protein (15 µg) were separated by SDS-PAGE (10%) and transferred to a nitrocellulose membrane. The phosphorylated and total non-phosphorylated proteins were detected using monoclonal antibodies to ERK1/2, p38, JNK and Akt. Proteins were visualized using with horseradish peroxidase-conjugated IgG anti-rabbit (1∶1,000 dilution, 1 h) and the ECL system. The resulting autoradiograms were analyzed with the AlphaEaseFC™ software V3.2 beta (Alpha Innotech).

### mRNA expression

RNA was isolated using TRIzol reagents (Life-Technologies). For the real-time reverse-transcriptase polymerase chain reaction (PCR), cDNA was synthesized using the RevertAidTM First Strand cDNA Synthesis Kit (Fermentas Life Sciences, Ontario, USA), according to the manufacturer's instructions. PCR-master mix (Power SyBr® Green, Applied Biosystems, Warrington, UK) containing the specific primers was then added. hCD36 forward: TCAGCAAATGCAAAGAAGGGAGAC and reverse: GGTTGACCTGCAGCCGTTTTG, IL-8 forward: ACTGAGAGTGATTGAGAGTGGAC and reverse: AACCCTCTGCACCCAGTTTTC, hMCP-1 forward: CAGCCAGATGCAATCAATGCC and reverse: TGGAATCCTGAACCCACTTCT and GAPDH forward: GAGTCAACGGATTTGGTCGT and reverse: TTGATTTTGGAGGGATCTCG. Real-time PCR was performed using a Stratagene Mx3005PTM QPCR Systems (Santa Clara, CA, USA). Relative gene expression was calculated by the 2^−Delta Delta C(T)^ method as previously described [32]. Data are shown in fold increase related to untreated cells.

### MTT assay

The mitochondrial-dependent reduction of methylthiazolyldiphenyl-tetrazolium bromide (MTT) to formazan insoluble crystals was used to evaluate cell viability. Briefly, 500 µg/ml of MTT in RPMI were added to the cells after the treatments. Cells were incubated for 3 h at 37°C in a 5% CO_2_ atmosphere. After that a SDS 10% in HCl 0.01 M solution was added to the cells to dissolve the crystals and the absorbance was measured after 14 h in a Dynatech microplate reader at 570 nm.

### Transfection of HEK 293t cells

HEK 293t cells were cultured in RPMI-1640 supplemented with 10% FCS, 15 mM HEPES, 2 mM L-glutamine, 100 U/mL penicillin and 100 µg/mL streptomycin. The plasmids hCD36-pcDNA3.2/V5-DEST, pcDNA3-kozac-myc-hPAFR and the control plasmid pcDNA3 were purified using the Qiagen Plasmid Kit. The day before the transfection, HEK 293t cells were seeded onto 24-well plates at a density of 2×10^5^ cells/well. The cells were transiently transfected with 2 µg of total plasmid DNA per well Lipofectamine-2000 according to the manufacturer's instructions. At 48 h after transfection, the cells were used for the experiments. The efficiency of the transfection was assessed by flow cytometry using PE-conjugated anti-human CD36 and by western blot using rabbit anti-hPAFR antibody.

### Measurement of IL-8 secretion

The IL-8 present in the supernatant of PMBC and HEK 293 transfected cells was evaluated by ELISA (BD Biosciences).

### Statistical analysis

Data are presented as mean ± SEM. Analysis of variance (ANOVA) and Student-Newman-Keuls post-test were used to evaluate the statistical significance of the differences. Significance was assumed if p<0.05.
